# SmTRC1, a novel *Schistosoma mansoni *DNA transposon, discloses new families of animal and fungi transposons belonging to the CACTA superfamily

**DOI:** 10.1186/1471-2148-6-89

**Published:** 2006-11-07

**Authors:** Ricardo DeMarco, Thiago M Venancio, Sergio Verjovski-Almeida

**Affiliations:** 1 Laboratory of Gene Expression in Eukaryotes; Departamento de Bioquímica, Instituto de Química, Universidade de São Paulo, Brazil; 2 Laboratory of Bioinformatics; Departamento de Bioquímica, Instituto de Química, Universidade de São Paulo, Brazil

## Abstract

**Background:**

The CACTA (also called En/Spm) superfamily of DNA-only transposons contain the core sequence CACTA in their Terminal Inverted Repeats (TIRs) and so far have only been described in plants. Large transcriptome and genome sequence data have recently become publicly available for *Schistosoma mansoni*, a digenetic blood fluke that is a major causative agent of schistosomiasis in humans, and have provided a comprehensive repository for the discovery of novel genes and repetitive elements. Despite the extensive description of retroelements in *S. mansoni*, just a single DNA-only transposon belonging to the Merlin family has so far been reported in this organism.

**Results:**

We describe a novel *S. mansoni *transposon named SmTRC1, for *S. mansoni *Transposon Related to CACTA 1, an element that shares several characteristics with plant CACTA transposons. Southern blotting indicates approximately 30–300 copies of SmTRC1 in the *S. mansoni *genome. Using genomic PCR followed by cloning and sequencing, we amplified and characterized a full-length and a truncated copy of this element. RT-PCR using *S. mansoni *mRNA followed by cloning and sequencing revealed several alternatively spliced transcripts of this transposon, resulting in distinct ORFs coding for different proteins. Interestingly, a survey of complete genomes from animals and fungi revealed several other novel TRC elements, indicating new families of DNA transposons belonging to the CACTA superfamily that have not previously been reported in these kingdoms. The first three bases in the *S. mansoni *TIR are CCC and they are identical to those in the TIRs of the insects *Aedes aegypti *and *Tribolium castaneum*, suggesting that animal TRCs may display a CCC core sequence.

**Conclusion:**

The DNA-only transposable element SmTRC1 from *S. mansoni *exhibits various characteristics, such as generation of multiple alternatively-spliced transcripts, the presence of terminal inverted repeats at the extremities of the elements flanked by direct repeats and the presence of a Transposase_21 domain, that suggest a distant relationship to CACTA transposons from Magnoliophyta. Several sequences from other Metazoa and Fungi code for proteins similar to those encoded by SmTRC1, suggesting that such elements have a common ancestry, and indicating inheritance through vertical transmission before separation of the Eumetazoa, Fungi and Plants.

## Background

Transposable elements constitute a large portion of the genomes of eukaryotes and play an important role in genome structure and evolution [1,2]. They can be assigned to two broad groups, retroelements (Class I) and DNA-only transposable elements (Class II). Unlike the retroelements, DNA-only transposons do not rely on a RNA intermediate, but transpose directly from DNA using a multi-step cut and paste mechanism catalyzed by a transposase that recognizes the transposon DNA by its Terminal Inverted Repeats (TIRs) (Reviewed in [3]). Class II elements in eukaryotes can be categorized on the basis of sequence similarities into nine superfamilies: Mariner-TC1; hAT; P; Mutator; CACTA; PIF/Harbinger; Transib; *piggyBac*; and Merlin [3-5].

Most elements of the CACTA superfamily of DNA-only transposons (also called En/Spm) contain the core sequence CACTA in their TIRs and so far have only been described in plants [3,5]. The prototypical CACTA maize Suppressor-mutator (Spm) transposon was one of the first transposons described by Barbara McClintock [6], and subsequent studies have shown that most of its length is occupied by a single transcription unit, which can be alternatively spliced to generate four distinct transcripts (*tnpA *to *tnpD*) encoding different proteins [7]. Two of these transcripts, *tnpA *and *tnpD*, encode proteins that have been shown to be essential for transposition [8,9]. TNPA protein has been shown to perform a role in reactivating the methylated transposon promoter and to repress active unmethylated promoter [10,11], while TNPD protein interacts directly with TNPA and stabilizes its binding to DNA [12].

*Schistosoma mansoni*, a digenetic blood fluke, is a major cause of schistosomiasis in humans and an important source of morbidity on a global scale. The disease is endemic in 74 developing countries, infecting about 200 million individuals, and an additional 500–600 million are estimated to be at risk [13]. The *S. mansoni *genome is approximately 270 Mbp long [14] and 55% of its content is expected to comprise mobile elements or other repetitive sequences [15]. Recently, independent transcriptome and genome sequencing initiatives have provided an extensive repository for the discovery of novel genes and repetitive elements [16,17]. Despite the extensive description of retroelements in *S. mansoni *[15,18,19], so far the presence of just a single DNA-only transposon, belonging to the Merlin family, has been reported in this organism [4].

Using the public repository of *S. mansoni *sequence data as a starting point, we describe a novel *S. mansoni *transposon named SmTRC1, an element that shares several characteristics with plant CACTA transposons, which suggests a distant relationship between these elements. A survey of complete genomes from the Animal and Fungi kingdoms revealed novel families of DNA transposons belonging to the CACTA superfamily.

## Results and discussion

### Isolation of SmTRC1 clones from *S. mansoni *genome

**Figure 1:**
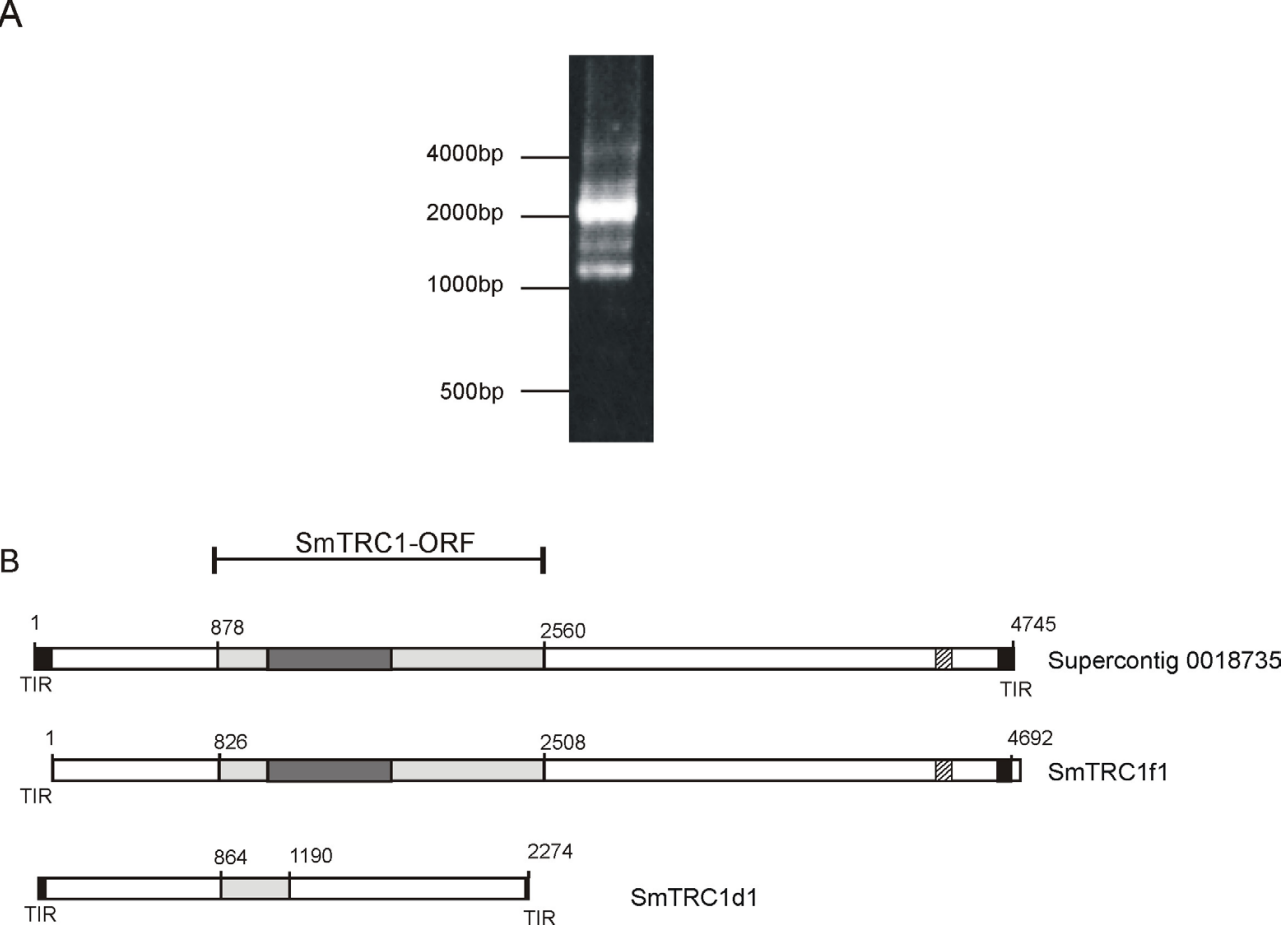
**SmTRC1 elements**. **A: **Agarose gel electrophoresis of typical PCR amplification products of *S. mansoni *genomic DNA with primers designed from the sequence of SmTRC1 extremities. **B: **Schematic representation of the SmTRC1 element derived *in silico *from *S. mansoni *shotgun genomic sequencing and assembly data obtained from the Sanger Institute (Supercontig 0018735) or from direct sequencing of clones amplified by PCR from genomic DNA obtained in this work (SmTRC1f1 and SmTRC1d1). Black boxes indicate the Terminal Inverted Repeats (TIR). Light gray boxes indicate the predicted SmTRC1-ORF and the dark gray box indicates the Transposase_21 domain within this ORF. The hatched box indicates a region with tandem repeats.

**Figure 2:**
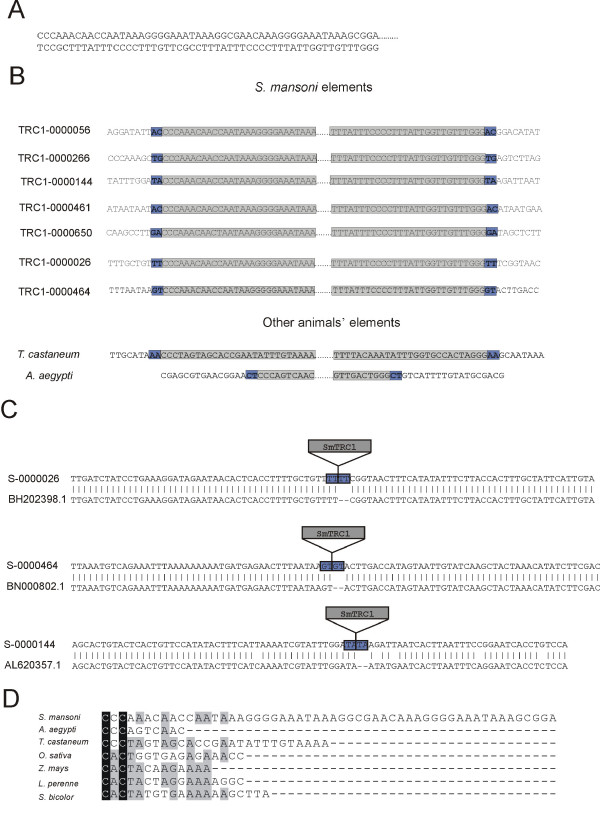
**Transposon inverted and direct repeats**. **A: **the complete sequence of SmTRC1 TIR is shown in this panel. Dots represent the transposon sequence not shown in the figure. **B: **blue boxes show direct repeats flanking *S. mansoni *and other animal TIRs (in gray). Only part of the *S. mansoni *TIR is represented in the figure. The dots represent the transposon sequence not shown in the figure. **C**: examples of target-site duplication created upon SmTRC1 insertion. Examples of alignments of sequences flanking SmTRC1 insertions (S-0000026, S-0000464 and S-0000144) with paralogous genomic sequences lacking transposon insertions (BH202398.1, BN000802.1 and AL620357.1) that were found in the *S. mansoni *public sequences database. The paralogous "gap" sequence (marked as –) presumably corresponds to the genomic target sequence before a transposon insertion event. Blue boxes indicate the target-site duplication in the flanking sequence. The number on the side of each sequence represents the supercontig from which it was derived (in the case of transposon inserted sequences) or GenBank accession numbers (in the case of paralogous sequences). **D: **TIR sequences from diverse CACTA superfamily animal and plant elements. The regions with high and medium levels of identity among the sequences are shown as black and gray columns, respectively.

Our attention was drawn to the *S. mansoni *genomic sequence of Supercontig_0018735 available at the Wellcome Trust Sanger Institute [20] while we were manually examining the splicing pattern of the gene represented by transcript SmAE C610100.1 [16]. Three exons of the latter mapped to bases 2032 to 2160, 6905 to 6961 and 7001 to 7231 of Supercontig_0018735. Upon examining the intron formed between bases 2160 to 6905 we detected a 4.5 kbp element that extended between bases 2164 and 6907 with one Open Reading Frame (referred to as SmTRC1-ORF in the following text) of 1,683 bp, which codes for a sequence with similarity (E-value 10^-5^) to a DNA-only transposon. An inverted repeat motif was found at both extremities (the left with 54 bp and the right missing only one base), suggesting that this intron is an inserted mobile element (Figure 1B). Although the element may be considered large (4.5 kpb) in comparison, for example, to Mariner family transposons (1.3 to 2.4 kpb) [21], it is considerably smaller than CACTA elements such as Spm/En (8.3 kpb) [8] and Rim coding elements (14.1 kpb on average) [22]. We detected several other copies of the element in the *S. mansoni *genome sequence dataset displaying a perfect 54 bp inverted repeat at both extremities, confirming the sequence and correct length of the Transposon Inverted Repeat (TIR) (Figure 2A). We named these elements SmTRC1 (an abbreviation for ***S****chistosoma ****m****ansoni ***T**ransposon **R**elated to **C**ACTA transposons) because of its several similarities to transposons of the CACTA family, as described in the text below.

We found a 2 bp direct repeat suggestive of target site duplication flanking the inverted repeat in 7 out of 14 (50%) of the copies of these elements analyzed (Figure 2B). Three of these copies were found to be inserted into repetitive elements of the *S. mansoni *genome. Two of them were inserted into copies of the *S. mansoni *LTR retrotransposons Saci-1 and Saci-4 [18,19] and one into an unidentified repetitive element of which there are at least 40 copies in the preliminary assembly of the *S. mansoni *genome. When the flanking sequences of these three elements were aligned with paralogous sequence copies of the respective repetitive element [GenBank:BH202328.1, GenBank:BN000802.1, GenBank:AL620357.1] obtained from either the GSS or the nr databases at GenBank, it was clear that the original repetitive element (not having an inserted transposon) contained none of the direct repeats flanking the SmTRC1 elements (Figure 2C); one of the 2bp direct repeat motifs was missing from the original repetitive element. This suggests that the direct repeats are in fact target-site duplications created by insertion of the SmTRC1 element. It is well known that transposon integration results in the duplication of a short host sequence at the insertion site and that the length of the target-site duplication is determined by the properties of each transposase [3], therefore these data provide further evidence for the mobility of SmTRC1 elements.

Using different combinations of a set of primers designed from the extremities of the transposon sequence from Supercontig_0018735, we performed several PCR reactions to amplify genomic copies of SmTRC1. A typical result is shown in Figure 1A; the major products are approximately 2.5 kbp. Cloning and sequencing of this major band revealed a 2274 bp clone that represented a truncated copy of SmTRC1, which we designated SmTRC1d1 (Figure 1B, bottom). This element displays a truncated ORF that codes for only 98 amino acids out of the 560 deduced from the full-length transposon. The entire sequence of SmTRC1d1 aligns with the full-length genomic transposon, but it lacks part of the ORF and its 3' tandem repeat (Figure 1B), hence its short length. The diversity of lower molecular weight bands generated by PCR (Figure 1A) suggests that SmTRC1 copies of several sizes, differently truncated, must exist in the *S. mansoni *genome.

For one set of PCR amplifications with genomic DNA as template, we used a 16 bp region downstream from the left TIR as forward primer, and a sequence composed of 8 bp overlapping the 3'-end of the right TIR plus a 12 bp region immediately downstream as reverse primer. This primer set permitted a copy of approximately 4.7 kb to be amplified; this copy was cloned and sequenced (Figure 1B, middle). The sequence was named SmTRC1f1; it lacked 71 bp at its 5'-end, including the left TIR, owing to the design of the primer used in the amplification reaction, but otherwise it appears to represent an integral copy of SmTRC1 (Figure 1B, middle). In fact, this clone displays 99.8 % nucleotide identity (only 9 mismatches over 4675 nucleotides) with the element described in Supercontig_0018735, and one base in the left TIR is deleted in both sequences. This suggests that we had cloned from the PCR products a copy representing the sequence contained in Supercontig_0018735; the nine mismatched bases may have arisen from mutations generated either naturally in the field among the copies from individual parasites, or artificially from the *Taq *polymerase during the PCR amplification step.

### Identification of TRC transposons in other species

A BLASTP search against the nr database at GenBank was performed using the protein encoded by SmTRC1-ORF as query. The highest hits produced were with two proteins of unknown function from *Schistosoma japonicum *[GenBank:AAW24935.1] (with E-value 10^-20^) and *Anopheles gambiae *[GenBank:EAA01922.3] (with E-value 10^-9^). The next best hits (E-value 10^-5^) were with the TNPD proteins of CACTA transposons from *Oryza sativa*. It is worth noting that the region displaying similarity corresponds exactly to the Transposase_21 domain (Pfam 02992). In fact, as described below, global alignment of this region of the SmTRC1-ORF product with the Transposase_21 domains of CACTA transposons indicates several conserved residues.

The SmTRC1-ORF sequence was used as query to perform an additional TBLASTN search directly into several complete animal and fungal genomes using the Genomic Blast tool at NCBI. This search produced hits indicating high similarity (E-value 10^-88 ^to 10^-30^) between the deduced SmTRC1-ORF translated sequence and translated sequences from genomes of such diverse animals as *Strongylocentrotus purpuratus*, *Ciona intestinalis*, *Danio rerio *and *Aedes aegypti*. In all these cases, practically the whole protein was aligned, not only the Transposase_21 domain. The search against fungal genomes produced hits indicating moderate similarity (E-values 10^-16 ^to 10^-4^) between the deduced SmTRC1-ORF translated sequence and translated sequences from the genomes of e.g. *Rhizopus oryzae*, *Coprinopsis cinerea *and *Phanerochaete chrysosporium*. For several of the above organisms, multiple hits were generated in the TBLASTN searches against the genomic sequence, indicating that more than one copy must be present in their genomes.

As noted earlier, SmTRC1 has a 54 bp TIR sequence at its extremities, which is consistently present in all copies examined. Also, the *S. mansoni *TIR sequence has an internal repeat of the motif AAAGGGGAAATAAAG. A TRC element of approximately 5.2 kb was detected in the *Tribolium castaneum *genomic sequence [GenBank:AAJJ01002287.1], and displays at its extremities an inverted repeat of 27 bp with the sequence CCCTAGTAGCACCGAATATTTGTAAAA. In addition, we found a 10 bp inverted repeat (CCCAGTCAAC) flanking a TRC in an *A. aegypti *genomic segment [GenBank:AAGE02020512] that delimits a 9.2 kb element. Both elements have a 2 bp direct repeat adjacent to each inverted repeat (Figure 2B), suggesting that target site duplication occurred when the mobile elements were inserted, analogous to the situation in the *S. mansoni *genomic sequences.

It is interesting to note that the first 3 bases in the TIRs from the *T. castaneum *and *A. aegypti *elements are identical to those in the TIR of *S. mansoni *(Figure 2D), suggesting that animal TRCs may display a CCC core sequence in their TIRs analogous to the CACTA core sequence in TIRs from CACTA transposons. Comparison of these animal TIRs with those of the plant CACTA transposons showed a high similarity between the *T. castaneum *TIR core sequence and the CACTA core sequence of plants, with only one mismatched base (Figure 2D). No such level of similarity is found in *S. mansoni *or *A. aegypti *TIR core sequences, which display only 3 and 2 coincident bases, respectively. Owing to the low numbers of bases and examples involved, it is difficult to determine whether the matching bases in *T. castaneum *are coincidental or reflect an evolutionary process.

We have not been able to characterize the TIRs flanking the ORFs of TRC elements in organisms other than those described above, namely *S. mansoni*, *A. aegypti *and *T. castaneum*. In some animal species, the TIRs may have become unrecognizable because of mutations or have been lost through further recombination. Another possibility is that some of these elements may represent domesticated transposases [23-26], which no longer transpose but perform another cellular function instead. Further analyses of these animal elements are warranted to determine which is the case for each particular element.

### Estimation of the number of copies of *Sm*TRC1 by Southern blotting

**Figure 3:**
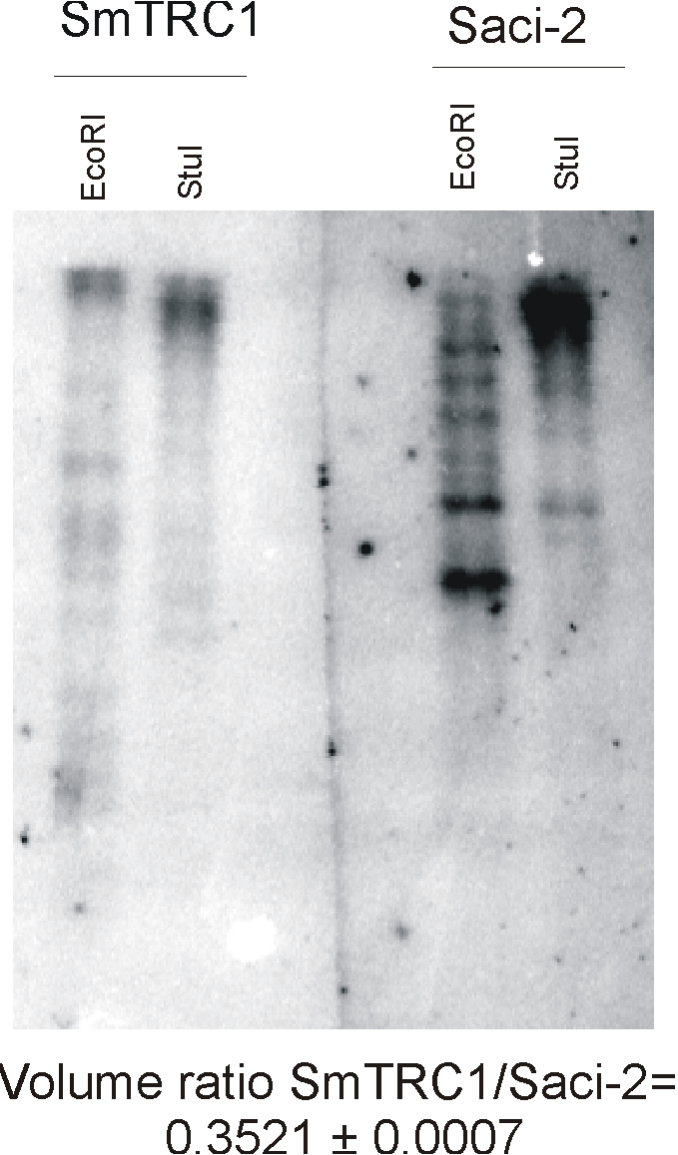
**Southern blotting of SmTRC1**. *S. mansoni *genomic DNA (5 μg) digested with the indicated restriction enzyme was loaded in each lane and analyzed by Southern blotting with a specific radiolabeled probe for SmTRC1. A parallel experiment was run with a probe for the Saci-2 retrotransposon [18], which was used as a benchmark. Probes of similar sizes and the same number of radioactive counts were used for each of the two hybridizations. Below the figure, the ratio between the total intensities of the SmTRC1/Saci-2 signals is indicated. This value was calculated for each digestion by integrating the signal from all the bands, and the average and total deviation was obtained by computing data from the two different digestions.

Southern blot analysis using a probe from the 5' end region of SmTRC1 detected multiple bands when hybridized to *EcoR*I-digested genomic DNA, showing the existence of multiple copies (Figure 3). In parallel experiments, we used the same number of radioactive counts and probes of approximately the same size for SmTRC1 and Saci-2, a previously described *S. mansoni *retrotransposon [18]. This allowed us to compare the two hybridization signals directly to estimate the number of SmTRC1 copies. The hybridization image was processed to measure the signal intensity in each lane. For each digestion, we divided the intensity from SmTRC1 by that from Saci-2. The results from both *EcoR*I and *Stu*I digestions show that SmTRC1 has approximately 1/3 of the number of copies of Saci-2 (estimated at 85–850 by DeMarco *et al*. [18]) (Figure 3). Extrapolation of these data indicates that there are approximately 30–300 copies of SmTRC1 in the *S. mansoni *genome. Amplification of SmTRC1 genomic DNA by PCR suggested that most of the genomic copies are not integral, implying that there are only a few full-length copies of SmTRC1 in the genome.

### SmTRC 1 produces multiple spliced transcripts

**Figure 4:**
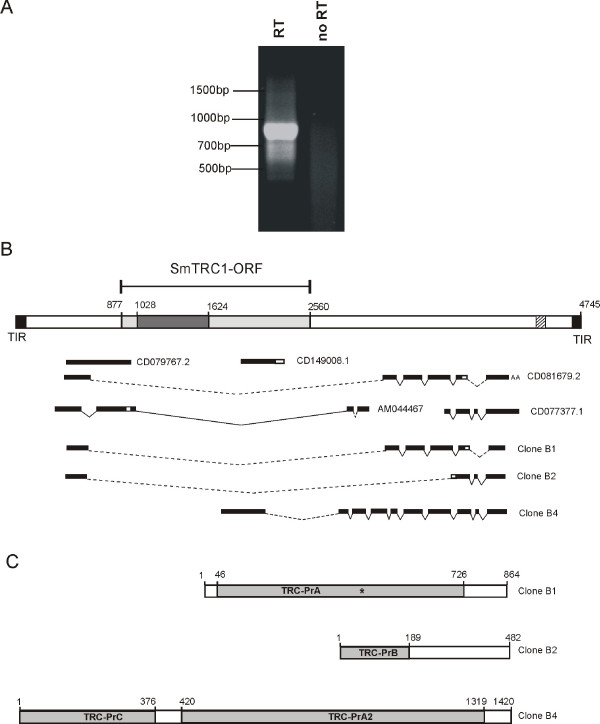
**Alternatively spliced forms of SmTRC1 transcripts**. **A: **agarose gel electrophoresis of products from an RT-PCR reaction with *S. mansoni *mRNA using primers designed from the sequence of the extremities of previously deposited ESTs mapping to the full-length SmTRC1 sequence. The "no RT" lane indicates a control in which no reverse transcriptase was added to the reaction medium. **B: **full-length SmTRC1 sequence (top scheme) and relative mapping positions of five existing ESTs from GenBank (Accession numbers shown next to each) and of three newly sequenced transcripts obtained by cloning the major band derived from the RT-PCR shown in panel A (Clones B1, B2 and B4 as indicated). Black boxes in the top scheme indicate the Terminal Inverted Repeats (TIR); light gray boxes indicate a predicted SmTRC1-ORF and the dark gray box indicates a Transposase_21 domain within this ORF; the hatched box indicates a region with tandem repeats. Thin black bars below the top scheme indicate mapped exons derived from each transcript; a white box indicates a region of a particular transcript not mapped to this specific copy of the SmTRC1 genomic sequence. Thin continuous lines represent junctions between interconnected exons in the transcripts, defining an intron with the canonical GT-AG splicing sites. Dashed lines represent junctions between interconnected exons in the transcripts, defining an intron without the canonical GT-AG splicing sites. Two "A"s indicate the presence of a poly-A tail. **C: **schematic representation of 3 clones of SmTRC1 transcripts. The scale in this part of the figure is expanded in comparison to that used in part B above. Light gray boxes indicate predicted ORF. Names inside the boxes indicate different hypothetical protein products coded by those transcripts. The asterisk indicates a stop codon present in the transcript but not in the equivalent genomic sequence of the full-length SmTRC1 element.

Mapping of the *S. mansoni *ESTs available at GenBank dbEST to the full-length genomic copy of SmTRC1 produced a discontinuous alignment of 64 ESTs (Figure 4B shows some representative examples), indicating that messages transcribed from this transposon are subject to alternative splicing. Moreover, several of the predicted introns contain the canonical GT-AG splicing sites at their extremities (Figure 4B). The diversity of patterns obtained for different ESTs mapping to the same *locus *indicates that a number of variant messages are produced by alternative splicing.

Intriguingly, no *S. japonicum *ESTs with homology to SmTRC1 were found by either BLASTN or TBLASTN searches using the full-length *S. mansoni *element as query against GenBank dbEST. This contrasts with the 70 *S. mansoni *ESTs (64 with discontinuous and 6 with continuous alignments) that were found with an E-value lower than 10^-10 ^by a BLASTN search against the same database. This leads us to hypothesize that these elements either have a much lower transcriptional activity in *S. japonicum *than in *S. mansoni *or have been eliminated from the *S. japonicum *genome.

To document the existence of alternatively spliced transcripts in *S. mansoni *better, we used the sequences at the extremities of many of the 70 ESTs known to align to SmTRC1 to design primers for amplifying additional SmTRC1 transcripts by RT-PCR using mRNA as template. The RT-PCR reaction produced a strong amplification band of approximately 900 bp and sub-products of lower molecular weight, plus a faint amplification band of higher molecular weight, which suggests that full-length transcripts may be expressed at a low level (Figure 4A). No amplification was seen in the control without reverse transcription, indicating that the amplification products were in fact derived from *bona fide *transcribed messages.

We cloned and sequenced these different RT-PCR products. Mapping the different sequences to the full-length SmTRC1 genomic sequence confirmed a diversity of splicing patterns, as already suggested by mapping of previously existing GenBank ESTs (Figure 4B). Two of the sequenced clones (Clones B1 and B2) are similar in size to the major band from the RT-PCR reaction, indicating that this band may contain more than one product. These two products did not overlap the ORF region, and the intron formed between their exons 1 and 2 does not display the canonical CT-AG splicing sites. However, mapping of these transcripts to the entire *S. mansoni *genome showed that canonical splicing sites are present at the extremities of the same intron in some truncated forms of the element (data not shown). This indicates that these transcripts probably originated from truncated copies that are apparently transcriptionally active, being responsible for a considerable fraction of the SmTRC1 transcripts detected.

Clone B1 has an ORF of 681 bp that is interrupted by a TGA stop codon at bases 439 to 441. However, alignment with the SmTRC1f1 sequence shows substitution of the TGA by CTA, which codes for isoleucine (Figure 4C). We named this hypothetical protein product SmTRC-PrA (*S. mansoni *TRC Protein A). In addition, a 4 bp deletion at base 37 of this clone in relation to SmTRC1f1 produces a frame shift at the beginning of the message. This suggests that the transcript was generated from a truncated copy of this element and that degeneration has produced the stop codon interrupting the ORF. Clone B2 has a shorter ORF that codes for a product very similar to the deduced N-terminal amino acids of the ORF product of clone B1; this hypothetical protein product was named SmTRC-PrB.

Although most of the ESTs exhibit a splicing pattern that does not include the SmTRC1-ORF sequence in any exon, the presence of a few ESTs mapped in the region of the SmTRC1-ORF suggests that it is actually transcribed and translated. We were not able to clone the high molecular weight products directly from the first RT-PCR using primers designed from the extremities of EST sequences, but we designed a primer from the 5'-end of the SmTRC1-ORF sequence and used a primer from the 3' extremity of the transcripts. The ensuing RT-PCR resulted in the amplification of a partial transcript of 1.3 kb (data not shown). Cloning and sequencing of this message showed that it was indeed derived from exons mapping to the SmTRC1-ORF as well as from other exons in the 3' region (Figure 4B, clone B4). It codes for an incomplete hypothetical protein product of 125 amino acids named SmTRC-PrC. A longer, complete version of the message encoding a longer SmTRC-PrC protein is expected to exist, including the Transposase_21 domain in its amino-terminal portion. Clone B4 also exhibits a second ORF coding for a protein of 299 amino acids, 175 of which are shared with SmTRC-PrA (76% of the SmTRC-PrA amino acids). In view of this level of conservation, we named this protein SmTRC-PrA2. Characterization of additional clones could eventually identify further alternatively spliced forms of SmTRC1-derived transcripts.

The Spm element of Maize has been shown to display four different transcripts (*TnpA-D*) generated by alternative splicing, each coding for a different protein product [7]. One of these transcripts (*TnpD*) spans the entire region comprising ORFs 1 and 2 predicted in the *spm *DNA, which explains the selection pressure that maintains such ORFs in the transposon structure. Similarly, we predict the existence of a spliced transcript spanning all the TRC1-ORF to explain the maintenance of such a large ORF and the conserved amino acid sequence observed on comparison with elements from other animals. The diverse splicing pattern of the transcribed *S. mansoni *messages results in distinct ORFs coding for different proteins; only the Transposase_21 domain of proteins encoded by these transposons has a detectable similarity with proteins from CACTA transposons. The other portions of the proteins encoded by these alternatively spliced *S. mansoni *transcripts appear not to be detectably conserved.

Spm *TnpA*, a short alternatively spliced transcript that lacks the Transposase_21 domain, is apparently more abundantly transcribed in plants than the other, longer transcripts. This reflects the results of RT-PCR experiments suggesting that shorter TRC transcripts are more abundantly transcribed in *S. mansoni *than the longer alternatively spliced transcript that includes the Transposase_21 domain. *Spm TnpD *displays two ORFs in tandem, one coding for TNPD and the other for TNPA [8]. Likewise, clone B4 also exhibits two ORFs in tandem, one coding for SmTRC-PrC and the other for a SmTRC-PrA-like protein.

### SmTRC 1 ORF contains a conserved Transposase_21 domain

**Figure 5:**
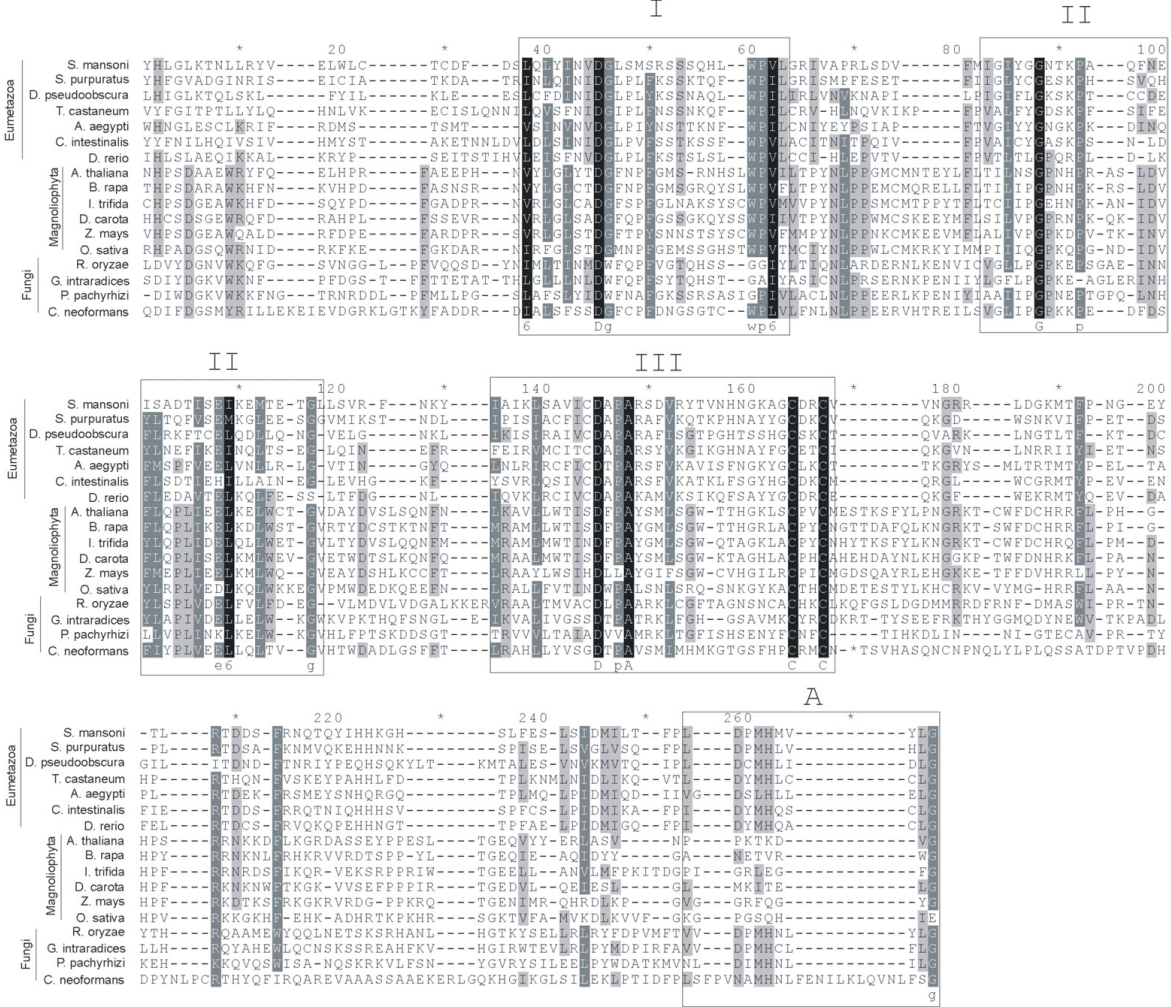
**Multiple alignment of the Transposase_21 domains of proteins of CACTA related transposons from diverse organisms**. Typical plant CACTA transposon sequences from six Magnoliophyta were included in the alignment. In addition, eleven novel CACTA-related elements identified here were included: seven from Eumetazoa and four from Fungi. Shading indicates the level of conservation of each residue. Boxes with Roman numbers I to III indicate conserved motifs of the Transposase_21 domain in all organisms. Box marked with A indicates a Transposase_21 motif displayed only by Eumetazoa and Fungi proteins.

Multiple protein sequence alignment (Figure 5) of the Transposase_21 domain (PFAM# PF02992) was performed using sequences from known CACTA transposons from several plants [22,27,28], together with the related domain in the deduced ORFs from *S. mansoni *and several other metazoan and fungal elements identified in the present work by BLAST analysis (as described above). Although there is a visible divergence between the domains from elements derived from different phyla, conservation of several residues is apparent (Figure 5). It is also interesting to note that the TRC-like sequence derived from the fungus *Cryptococcus neoformans *has several characteristics that distinguish it from the other fungal sequences, which are apparently very similar to one another.

Three different conserved motifs can be discerned, marked I-III in the aligned proteins (Figure 5). In most of the elements, a first conserved motif I/L/V-X-I/L/V/F-X-I/L/V/F-X_2_-D-G-X_3_-F/Y-X_7–9_-W-P-I/L/V of Transposase_21 domain is present (Figure 5, box I); however, in 3 out of 4 fungal sequences this motif shows an interchange between tryptophan and glycine residues. This suggests that these two residues have an important role and that only the position of one residue relative to the other is essential for activity of this protein. In the second conserved domain (Figure 5, box II), the fungal proteins show a level of conservation comparable to Magnoliophyta and higher than Eumetazoa. Thus, only proteins from Fungi and Magnoliophyta have a proline immediately after the conserved glycine residue (Figure 5, box II), and an additional P-L/I conserved motif in the middle of this domain; both are absent from Eumetazoa. Interestingly, we identified a L/V/I-D-X-L/M-H-X_3_-L-G motif in the Transposase_21 domain that is present in the eumetazoan TRC elements (Figure 5, box A) and also in 3 out of the 4 fungal TRC proteins, but is absent in proteins from Magnoliophyta.

Among the conserved residues there are two aspartyl residues (DD), one in the first and one in the third conserved domain, separated by 80 residues; this is very similar to the distance between the two aspartyl residues in the DDE motifs in Tn3 transposases [29]. There is also a glutamyl residue (E) in the conserved domain 2 in all but three sequences, two of which have this residue in adjacent positions and one in a position 2 residues away. It is possible that conservation of such an amino acid triad reflects a similar catalytic mechanism in the DDE motif despite the different arrangement of residues. In this case its function would be similar to that described for the DDE motif, which is presumed to coordinate divalent metal ions to promote catalysis of DNA cleavage and ligation [3]. Moreover, there is a conserved CXXC motif in the conserved domain 3 that is identical to the configuration of cysteines in the zinc-finger-like motifs (HHCC domains) of retroviral integrases, suggesting that TRC cysteines may also be involved in DNA binding.

**Figure 6:**
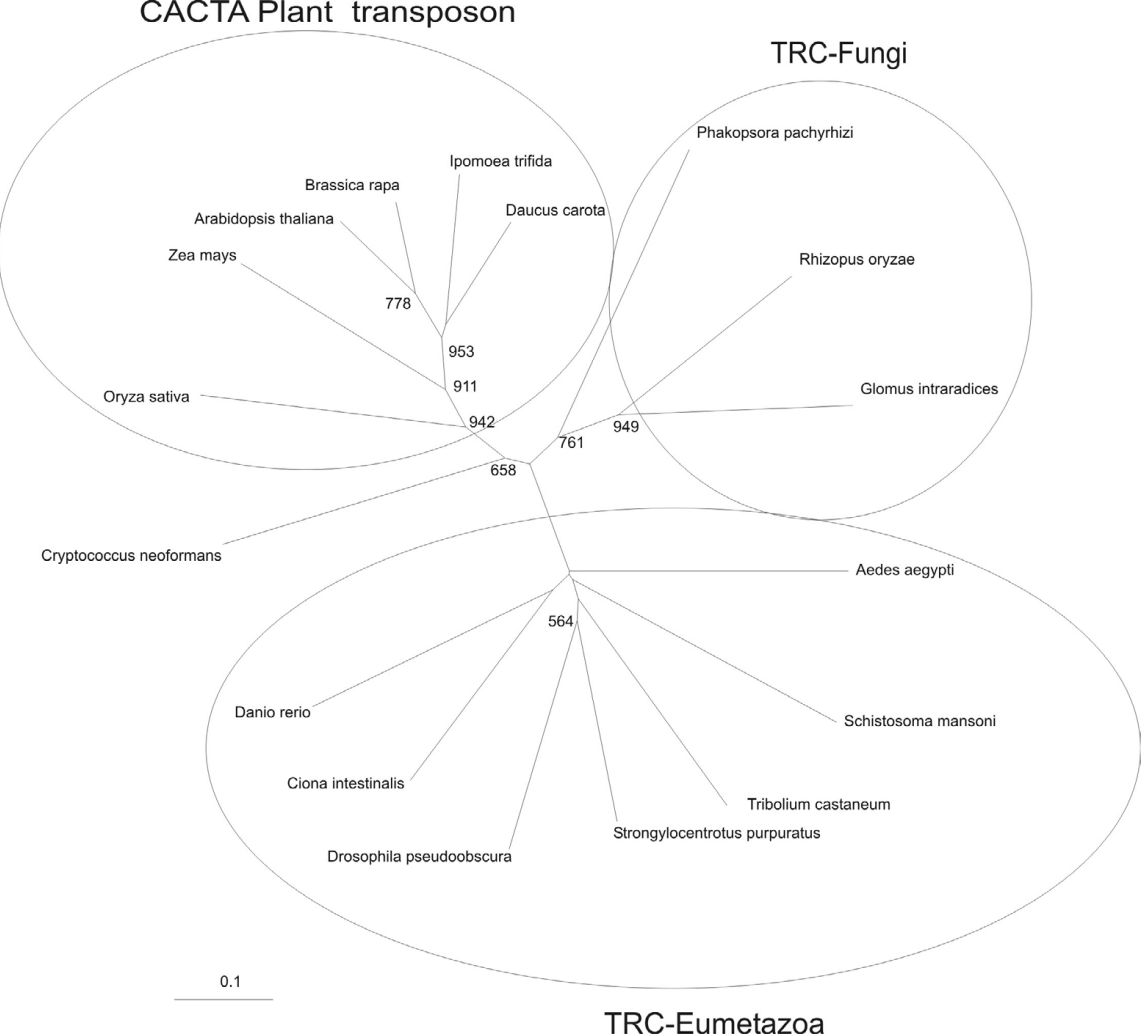
**Phylogenetic tree for the Transposase_21 domains of CACTA-like transposases**. The tree was constructed by the neighbor-joining method using the three conserved regions indicated by boxes I to III in Figure 5 and excluding positions with gaps. Numbers represent the confidence of the branches assigned by bootstrap analysis (in 1,000 samplings); bootstrap values lower than 500 are omitted from the figure. The names indicate a transposon member of the CACTA or of the Transposon Related to CACTA (TRC) family belonging to the organism indicated. Circles indicate the 3 different proposed families of transposons within the CACTA superfamily.

A phylogenetic tree (Figure 6) was generated from the three conserved regions (I-III) shown in the alignment of Figure 5. Although the analysis does not permit a clear inference of phylogeny within the Eumetazoan elements, it clearly shows the separation of the Eumetazoa and Plant branches (Figure 6). Interestingly, three of the Fungi elements appear to be distinct from the others, but one of them, the *C. neoformans *element, appears to be at a basal position in relation to plant transposons. With the exception of the latter, the analysis clearly shows the separation between Plants, Fungi and Eumetazoa.

## Conclusion

The DNA-only transposable element SmTRC1 from *S. mansoni *exhibits various characteristics, such as generation of multiple spliced transcripts, the presence of terminal inverted repeats at the extremities of the elements flanked by direct repeats and the presence of a Transposase_21 domain, that suggest a distant relationship to CACTA transposons from Magnoliophyta. Despite these similarities, conservation of proteins deduced from this new family of transposons in relation to CACTA transposons is restricted to the Transposase_21 domain. The presence in *S. mansoni *of multiple transcripts and a higher expression level of a shorter alternatively spliced transcript coding for an ORF lacking the Transposase_21 domain suggests that similar strategies are employed by transposons from animals and plants. The absence of conservation between SmTRC-PrA/SmTRC-PrB and plant TNPA protein indicates that these *S. mansoni *proteins either have a different evolutionary origin or are very divergent. The latter is more probable since we could detect no similarity between the *S. mansoni *proteins SmTRC-PrA and SmTRC-PrB and any sequences encoded in other animal or fungal TRC elements by BLASTP or TBLASTN comparison to the genomes of these organisms, suggesting that this protein is rapidly evolving. Nevertheless, SmTRC-PrA must perform an analogous role to the DNA binding function described for TNPA in plant transposons, and experimental verification of this hypothesis is warranted.

Several sequences from Metazoa and Fungi code for proteins similar to those encoded by SmTRC, providing evidence that this superfamily exists in branches other than Plants. Data from phylogenetic analysis of the Transposase_21 domain suggests a common ancestry for such elements, and indicates inheritance through vertical transmission before the separation of Eumetazoa, Fungi and Plants. This organization permits a division of the CACTA superfamily into 3 different families, each represented by one of these branches (circles in Figure 6). The *C. neoformans *element appears to be an exception, being more closely related to the plant transposon family than to the Fungi family.

In view of the evolutionary distance between these related elements, the few conserved amino acids of the Transposase_21 domain must be essential for TNPD function. These conserved residues are preferential targets for future mutagenesis experiments to determine the importance of the Transposase_21 domain for TNPD function, as they are expected to abolish or significantly alter the domain functionality.

Discovery of this new transposable element in *S. mansoni *should help in obtaining a more complete annotation of the genome of this parasite. Apparently DNA transposons are not as widespread as retroelements in *S. mansoni*, since only 2 elements of the former type ([4]; this report) and 28 of the latter type [15,19] have been described. Nevertheless, DNA transposons may have a significant impact on the biology of the parasite. Comparison between Merlin and SmTRC1 shows that these elements have very distinct characteristics, the former being more compact (1.4 kpb [4] as opposed to 4.5 kbp) and presenting a slightly higher copy number, with an estimated 500 copies [4] compared to 30–300 for SmTRC1. On the other hand, most of the copies from both SmTRC1 and Merlin elements appear to be internal deletion derivatives. Several transcripts of both elements have been detected in the *S. mansoni *EST database, suggesting that both are transcriptionally active.

In addition, it is interesting to consider the SmTRC1 element as a potential new tool for insertional mutagenesis experiments in *Schistosoma *and other platyhelminths; CACTA elements have been successfully used for this purpose in plants [30,31]. Moreover, other superfamilies of transposons have been widely used for invertebrate and vertebrate transgene experiments and constitute a valuable tool for analyzing gene function and effects on phenotype [32-34]. Further studies on other Metazoa and Fungi elements from the family described here will certainly provide candidate vectors for several other organisms.

## Methods

### RT-PCR and genomic DNA PCR

The BH isolates of *S. mansoni *were maintained in the laboratory by routine passage through mice and snails. Adult parasites were obtained by portal perfusion of hamsters 7 to 8 weeks after infection. Tissues were conserved in RNALater (Ambion) according to the manufacturer's instructions. Tissue mRNAs were extracted using MAC isolation kits (Miltenyi Biotec). mRNA samples were treated with RQ1 RNase-free DNase (1 U/10 μl; Promega) for 30 minutes at 37°C. cDNAs were prepared using the Superscript II first strand synthesis system for RT-PCR (Invitrogen), following the manufacturer's instructions. A parallel control reaction was performed without the addition of reverse transcriptase and used as template for a PCR reaction to detect any genomic DNA contamination. The PCR step was performed using Advantage II polymerase (Clontech) with the buffer supplied by the manufacturer, 200 μM dNTPs, and 200 nM of each primer using the following program: 95°C (1 min); 35 cycles of 95°C (30 s), 55°C (30 s) and 68°C (4 min); and final extension at 68°C (3 min). The reaction products were cloned into pGem-T vector (Promega) and sequenced.

Genomic DNA was extracted from 800 mg of adult worms (approximately 3,000 worms) using the protocol described by Ausubel et al. [35]. PCR reactions were performed using the same conditions as described in the previous paragraph.

### Southern Blot

Southern blot experiments for estimating the number of copies of SmTRC1 were performed according to the protocol described by DeMarco et al. [18], except that *Stu*I was used in place of *BamH*I and that Saci-2 was used as a benchmark. The total signal intensity for each lane was calculated using ImageQuant v5.1 (Molecular Dynamics), with a fixed area rectangle to delimit the area for each sample analyzed.

### Sequence alignment and construction of phylogenetic trees

Using the deduced protein sequence of the Transposase_21 domain from SmTRC1, we retrieved several other sequences that showed significant similarity (E-values less than 10^-4^) in a tBLASTn search against genomes of Metazoa and Fungi at NCBI. We used these sequences along with the sequences of known CACTA transposons from Magnoliophyta to perform an alignment using ClustalX v1.83 [36]. Alignments were imported to the GeneDoc program V2.6.002 for shading of conserved residues. Further analysis with Clustal X and the neighbor-joining method, using the three conserved regions indicated by boxes I to III in Figure 5 and excluding positions with gaps, resulted in the phylogenetic tree shown in Figure 6. The confidence of the branches was evaluated by bootstrap analysis using 1,000 samplings. Phylogenetic trees were drawn using Treeview (version 1.6.6) [37]. The GenBank sequences and accession numbers utilized for construction of alignments and phylogenetic trees are as follows: (1) Transposase family tnp2 members – *Arabdopsis thaliana*, [GenBank:CAB80813.1]; *Brassica rapa*, [GenBank:BAA85462]; *Daucus carota*, [GenBank:BAA20532.1], *Oryza sativa*, [GenBank:DAA02106.1]; *Zea mays*, [GenBank:AAA66266.1]; *Ipomoea trifida*, [GenBank:AAS79612.1]; (2) Whole genome DNA sequences – *Aedes aegypti*, [GenBank:AAGE02020512]; *Danio rerio*, [GenBank:CAAK02057130.1]; *Strongylocentrotus purpuratus*, [GenBank:AAGJ01221697.1]; *Ciona intestinalis*, [GenBank:AABS01001120.1]; *Tribolium castaneum*, [GenBank:AAJJ01002287.1]; *Drosophila pseudoobscura*, [GenBank:AADE01004520.1]; *Glomus intraradices*, [GenBank:AC156590]; *Phakopsora pachyrhizi*, [GenBank:AC149399]; *Cryptococcus neoformans*, [GenBank:EAL18770.1]; *Rhizopus oryzae*, [GenBank:AACW02000214.1].

### Accession numbers of sequences identified in this work

We have deposited all sequences obtained in this work at EMBL under the following numbers: SmTRC1f1, [EMBL:AM268206]; SmTRC1d1, [EMBL:AM268205]; SmTRC-PrA, [EMBL:AM268207]; SmTRC-PrB, [EMBL:AM268208]; SmTRC-PrC, [EMBL:AM268209]. We have also deposited Third Party Annotations (TPAs) at EMBL for Transposase family Tnp2 members, under the following TPA numbers: *Aedes aegypti*, [EMBL:BN000947]; *Danio rerio*, [EMBL:BN000951]; *Strongylocentrotus purpuratus*, [EMBL:BN000955]; *Ciona intestinalis*, [EMBL:BN000948]; *Tribolium castaneum*, [EMBL:BN000946]; *Drosophila pseudoobscura*, [EMBL:BN000950]; *Glomus intraradices*, [EMBL:BN000952]; *Phakopsora pachyrhizi*, [EMBL:BN000953]; *Rhizopus oryzae*, [EMBL:BN000954]; *Cryptococcus neoformans*, [EMBL:BN000949].

## Authors' contributions

RdeM conceived of the study, carried out the molecular genetic experiments, participated in the sequence alignment and drafted the manuscript. TMV participated in the sequence alignment. SVA participated in the design of the study and coordination and drafted the manuscript. All authors read and approved the final manuscript.
